# Economic evaluation of bailing capsules for patients with diabetic nephropathy in China

**DOI:** 10.3389/fphar.2023.1175310

**Published:** 2023-07-05

**Authors:** Yumei He, Wei Li, He Zhu, Sheng Han

**Affiliations:** ^1^ International Research Center for Medicinal Administration, Peking University, Beijing, China; ^2^ School of Pharmaceutical Sciences, Peking University, Beijing, China

**Keywords:** bailing capsules, diabetic nephropathy, cost-effectiveness analysis, Chinese population, markov model

## Abstract

**Background:** Diabetic nephropathy is a major microvascular complication and the main cause of end-stage renal disease in diabetic patients. The therapeutic effects of Bailing capsules for diabetic nephropathy have already been demonstrated; however, the cost-effectiveness of Bailing capsules remains controversial. This study aimed to evaluate the cost-effectiveness of Bailing capsules combined with Western medicine compared with Western medicine alone in diabetic nephropathy from a Chinese healthcare system perspective.

**Methods:** A Markov model was established to simulate the disease process of patients over a 20-year period. Clinical efficacy data were obtained from a meta-analysis, and transition probability was estimated based on microsimulation. Direct costs and utility values were collected from the Chinese Drug Bidding Database (https://www.shuju.menet.com.cn) and published literature. The incremental cost-effectiveness ratio (ICER) was measured, and one-way and probabilistic sensitivity analyses were performed to observe model stability.

**Results:** A total of 34 randomized controlled trials involving 3,444 patients with diabetic nephropathy were selected for the meta-analysis. Compared to Western medicine alone, the addition of Bailing capsules resulted in an increase of 0.39 quality-adjusted life-years (QALYs) and additional costs of Chinese Yuan (CNY) 24,721, yielding an ICER of CNY 63,001 per QALY gained. The ICER was lower than the threshold of willingness-to-pay of CNY 80,976 (The GDP per Capita in China). The reliability and stability of the results were confirmed by the sensitivity analysis.

**Conclusion:** We found that Bailing capsules may be a cost-effective treatment choice for patients with diabetic nephropathy in the Chinese population.

## Introduction

The International Diabetes Federation indicates that currently, 425 million people suffer from diabetes worldwide, an incidence that will increase to 629 million by 2045 without effective control (IDF, 2019). Diabetic nephropathy is a serious chronic microvascular complication induced by long-term hyperglycemia in diabetic patients and is regarded as the most prevalent cause of chronic kidney disease (Doshi and Friedman, 2017; American Diabetes Association Professional Practice Committee, 2022). Currently, diabetic nephropathy has overtaken glomerulonephritis-related chronic kidney disease (CKD) to become the most common cause of end-stage renal disease in China (Zhang et al., 2016). The pathogenesis of diabetic nephropathy is related to hyperglycemia and insulin resistance in diabetic patients and is caused by abnormal lipid metabolism, inflammation, and oxidative stress (Guo et al., 2020; Sagoo and Gnudi, 2020). However, no obvious manifestations of diabetic nephropathy in the early stages exist, owing to its insidious nature. For patients who reach the clinical stage of nephropathy and whose treatment of diabetic nephropathy is delayed, renal lesions are irreversible (Viigimaa et al., 2020).

The treatment of diabetic nephropathy aims to control blood glucose levels, reduce urine protein levels, and improve renal microcirculation (Flyvbjerg, 2017). However, the use of Western medicine for diabetic nephropathy is associated with long-term adverse events. Recently, Chinese medicine applications for the treatment of diabetic nephropathy have increased, aimed at controlling the symptoms and slowing its progression. Traditional Chinese medicine has the advantage of providing good curative effects with few side effects, while relieving symptoms (Zhao et al., 2018). The Bailing capsule contains *Cordyceps* polysaccharides and amino acids, which are refined by low-temperature fermentation of *Cordyceps* strains. Studies have indicated that Bailing capsules have antioxidative, anti-inflammatory, and proteinuria reducing effects (Yuan et al., 2018; Dong et al., 2019a; Liu et al., 2019). Previous meta-analyses found that Bailing capsules combined with Western medicine were superior to Western medicine alone in improving the symptoms of diabetic nephropathy, including 24 h total urine protein, urine albumin excretion rate, serum creatinine, and blood urea nitrogen (Luo et al., 2015; Huang et al., 2019; Sheng et al., 2020). Considering that the treatment of non-dialysis patients with diabetic nephropathy has significantly increased healthcare expenditures and patients requiring long-term treatment, evaluation of the cost-effectiveness of Bailing capsules is warranted. To date, no such studies have been published; thus, we proposed to assess the cost-effectiveness of Bailing capsules combined with Western medicine compared with Western medicine alone for patients with diabetic nephropathy based on clinical efficacy data from a meta-analysis, which could provide evidence to help choose the most cost-effective treatment regimen.

## Methods

### Meta-analysis

#### Literature search and selection criteria

The Preferred Reporting Items for Systematic Reviews and Meta-Analysis guidelines issued in 2020 were used to guide and report this study (Moher et al., 2009). The PubMed (https://pubmed.ncbi.nlm.nih.gov/), Embase (https://www.embase.com/), Cochrane library (https://www.cochranelibrary.com/), VIP (http://www.cqvip.com/), Wanfang (https://www.wanfangdata.com.cn/), and China National Knowledge Internet (https://www.cnki.net/) databases were systematically searched throughout the month of December 2021 for peer-reviewed literature, and the following terms were used, including MeSH terms and free keywords: “diabetic nephropathy” AND “Bailing capsule” AND “randomized controlled trials.” No restrictions were placed on publication language and status. The details of the search strategy in PubMed are shown in Supplementary Table S1. Additional potentially eligible studies were identified by manually reviewing reference lists and the clinicaltrials.gov website.

The literature search and study selection were independently performed by two reviewers, and conflicts between reviewers were resolved by group discussion until a consensus was reached. Study inclusion criteria were as follows: 1) patients, non-dialysis patients with diabetic nephropathy; 2) intervention, Bailing capsule combined with Western medicine; 3) control, Western medicine alone; 4) outcomes, serum creatinine; and 5) study design, randomized controlled trial (RCT) design. The exclusion criteria were as follows: 1) the intervention only indicated artificial *Cordyceps* preparations, while the generic name was not included; 2) the intervention regimens contained other Chinese medicine drugs; 3) the investigated outcomes, treatment duration, and baseline characteristics were incomplete; and 4) the study design was not mentioned.

#### Data extraction and quality assessment

Two reviewers independently extracted relevant information from eligible RCTs following a standardized flow, and disagreements between the reviewers were settled by discussing until a consensus was reached. The collected information included the first author’s surname, publication year, region, sample size, mean age, sex, duration of disease, intervention, control, dose of Bailing capsule, treatment duration, and reported outcomes. The risk of bias approach, according to the methods described by the Cochrane Collaboration, was used to assess the quality of the study, which included random sequence generation, allocation concealment, blinding of participants and personnel, blinding of outcome assessment, incomplete outcome data, selection of reporting, and other biases (Higgins et al., 2011). The quality assessment was evaluated by two reviewers, and any disagreement was resolved by an additional reviewer referring to the full text of the original article.

#### Statistical analysis

The efficacy of the Bailing capsule was evaluated in terms of serum creatinine, and weighted mean difference with a 95% confidence interval (CI) applied as an effect estimate. Heterogeneity across the included trials was assessed using *I* ^*2*^ and Q statistics (Higgins et al., 2003). The fixed-effect model was applied to calculate the pooled effect estimate if *p* > 0.10, and *I* ^*2*^ < 25%, while the random-effects model was used if *p* < 0.10 or *I* ^*2*^ > 25% (DerSimonian and Laird, 2015). Meta-regression analyses were performed to identify potential sources of heterogeneity on the basis of sample size, mean age, male proportion, duration of disease, and treatment duration. Quality assessment was performed using Review Manager version 5.3 from the Cochrane Collaboration and meta-analysis was performed using the STATA software (version 15.0; Stata Corporation, College Station, TX, United States).

### Economic evaluation

#### Patients and regimens

A hypothetical cohort that matched the inclusion criteria for the meta-analysis was incorporated into the model. These patients were non-dialysis patients with diabetic nephropathy. The treatment strategies assessed in the economic evaluation included: 1) Western medicine group: Western medicine alone, which aimed to control blood glucose, blood pressure, serum lipids, with no restrictions placed on treatment drugs or dosage; 2) Bailing capsules group: based on the treatment in the Western medicine group, with Bailing capsules added at a 2 g dose, three times per day.

#### Model structure

Considering that diabetic nephropathy is a CKD, a model was created in Microsoft Excel and structured as a Markov model according to the progression of CKD and the existing literature for economic assessment in the treatment of CKD. The Markov model diagram is presented in Supplementary Table S2. The model included five health states: CKD stage 3, CKD stage 4, CKD stage 5 (non-hemodialysis), CKD stage 5 (hemodialysis), and death. Patients’ initial state was CKD stage 3, and individuals could maintain CKD stage 3, progress from stage 3 to stage 4 or death. Patients at CKD stage 4 could maintain stage 4, progress from stage 4 to stage 5 or death. Some patients at CKD stage 5 were treated with hemodialysis, while some patients were not. Patients with CKD stage 5 could maintain CKD stage 5 or progress to death, and general disease progression was irreversible. Considering 89.1% of end-stage renal disease patients were treated with hemodialysis (Zhang and Zuo, 2016), this treatment was included in the study. Moreover, clinical experts have hypothesized that the effects of Bailing capsules mainly delay the progression of CKD stage 3 to stage 4 or CKD stage 4 to stage 5, while they have no significant effect on CKD stage 5 and hemodialysis patients, and no significant therapeutic difference between CKD stage 3 and stage 4 patients.

The time horizon was 20 years, the cycle length was 1 year, and a half-cycle correction (i.e., averaging outcomes between the beginning and end of each cycle to reflect that events can occur at any point within the cycle) was applied to all costs and outcomes. An economic evaluation was conducted from the perspective of the Chinese healthcare system. The outcome was measured in quality-adjusted life-years (QALYs) and total direct medical costs. The incremental cost-effectiveness ratio (ICER) was then calculated. The costs and benefits were discounted at 5.0% annually (in accordance with China Guidelines for Pharmacoeconomic Evaluations) (Liu, 2000). The GDP *per capita* in 2021 [Chinese Yuan (CNY) 80,976] was taken as the willingness-to-pay (WTP) threshold.

#### Model inputs

A microsimulation was performed to calculate the transition probability between various CKD stages. The glomerular filtration rate (GFR) is widely used to assess renal function, and it still shows a downward trend in patients receiving effective treatment (Zhang et al., 2012; Lai et al., 2014; Weng et al., 2014; Guan et al., 2017). The transition probabilities of CKD stage 3 to stage 4 and from CKD stage 4 to stage 5 were calculated based on the following: 1) the disease status was judged according to the GFR level; 2) the GFR was decreased during CKD stage 3 or after; and 3) the improvement in serum creatinine in patients at CKD stages 3 and 4 was similar. The rate of annual GFR decline was 1.7 mL/min/1.73 m^2^ (Orlando et al., 2011), and the GFR level after treatment was calculated using a modification of diet in renal disease equation (Eq. 1) according to the characteristics of the Chinese population (MDRD, 2013). The improvement of serum creatinine after treatment was obtained from the results of the meta-analysis, and sex or age was set based on the baseline characteristics of patients in the meta-analysis.

$$eGFR=186\times {Scr}^{-1.154}\times {\left(age\right)}^{-0.203}\left[\times 0.742\;\left(female\right)\right]\left[\times 1.233\;\left(Chinese\;population\right)\right]$$ (1)

A cohort of 10,000 patients was generated in the microsimulation and the age, sex, serum creatinine at various CKD stages, and annual reduction in GFR of individual patients were assumed as certain distribution types, which are listed in Supplementary Table S3. Mortality rates at various CKD stages were obtained based on published studies.

The cost of Bailing capsules per cycle was calculated as follows: drug cost = unit price of drug × daily dosage × cycle × medication compliance. The price of the Bailing capsules was CNY 43.26 (0.5 g × 42 tablets); the daily dosage of Bailing capsule was 12 tablets per day (0.5 g/tablet). The Western medicine and hemodialysis costs were obtained from published articles. Medication compliance was 92.75%, according to a previous study (Chen et al., 2011). Utility values were obtained from the literature using the Health Utilities Index Mark 3 scale (Gorodetskaya et al., 2005). Considering the low prevalence and mild symptoms of adverse events and that no additional treatments were applied for adverse events, the treatment cost for adverse events and their impact on quality of life were not addressed in the model. The details of the Markov model parameters are presented in Supplementary Table S4.

#### Sensitivity analysis

One-way and probabilistic sensitivity analyses (PSA) of the model parameters were performed to assess the robustness of the evaluation model. The results of the one-way sensitivity analysis were represented by a tornado diagram. The range of each parameter used in the one-way sensitivity analyses was based on either the 95% CI reported in the referenced literature or a ±30% change from the base case value.

In the PSA, all costs were assigned with a gamma distribution and probability, proportion, and utilities were assigned with a beta distribution, extracting the values of the corresponding distribution for 1,000 Monte Carlo simulations. The PSA results are represented by a cost-effectiveness acceptability curve.

## Results

### Meta analysis

#### Search of the literature

**FIGURE 1:**
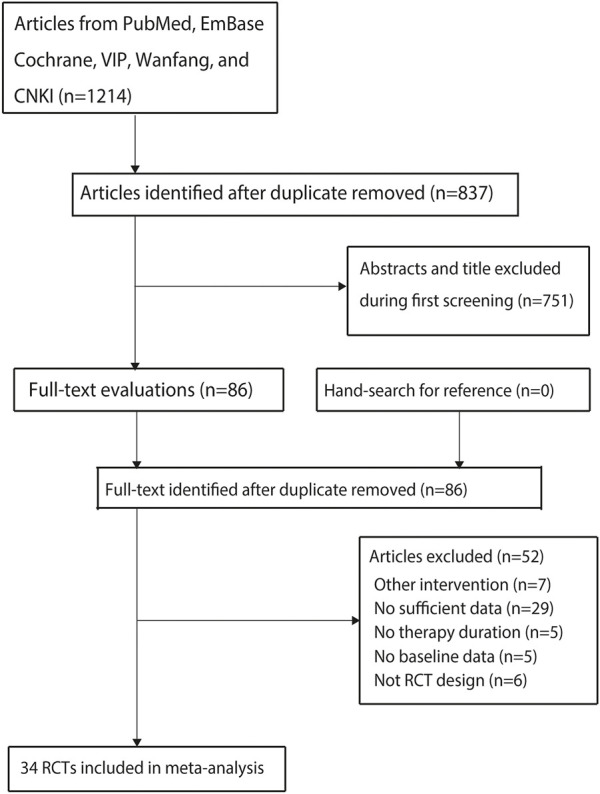
The flowchart for the process of literature search and study selection.

A total of 1,214 articles were identified from the initial search in the electronic databases, and 837 studies were retained after duplicate articles were removed. An additional 751 studies were removed due to irrelevant titles or abstracts in the initial screening, and the remaining 86 studies were retrieved for full-text evaluation. Then 52 studies were excluded for the following reasons: 1) other interventions (*n* = 7); 2) insufficient data for serum creatinine (*n* = 29); 3) no reported treatment duration (*n* = 5); 4) no reported baseline characteristics (*n* = 5); and 5) no RCT design (*n* = 6). The remaining 34 studies were selected for the final meta-analysis (Luo and Hu, 2011; Wang, 2011; Zong et al., 2014; Tang et al., 2015; Chen et al., 2016; Gao et al., 2016; Hu, 2016; Jin, 2016; Li et al., 2016; Lin, 2016; Peng et al., 2016; Wang et al., 2016; Yang, 2016; Yang and Liu, 2016; Tang et al., 2017; Gao, 2018; Hu et al., 2018; Luo et al., 2018; Wang, 2018; Wu, 2018; Yang et al., 2018; Dong et al., 2019b; Tian, 2019; Zhou, 2019; Cheng et al., 2020; Niu, 2020; Ren and Hu, 2020; Shi, 2020; Yang, 2020; Zhan, 2020; Zhang, 2020; Zhong et al., 2020; Guan et al., 2021; Li and Gao, 2021). No additional eligible trials were identified from the manually reviewed reference lists of the original articles (Figure 1).

#### Characteristics of the included studies

**FIGURE 2:**
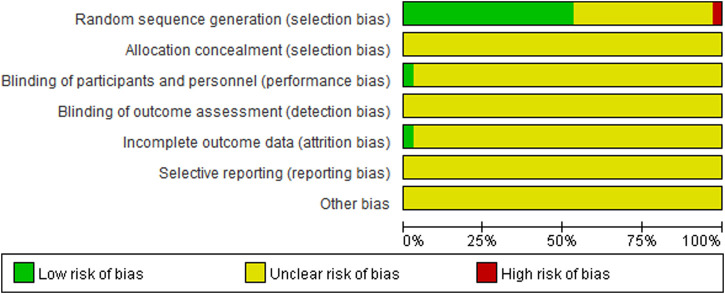
The overall quality of included trials.

The baseline characteristics of the RCTs are presented in Table 1. The 34 included trials comprised 3,444 patients with diabetic nephropathy, with sample sizes ranging from 62 to 170. The treatment duration ranged from 2 weeks to 6 months. The quality of the included trials is summarized in Figure 2 and Table 2. Of the included trials, 15 mentioned randomization; however, these trials did not report the generation of random sequences. None of the studies mentioned allocation concealment, blinding of outcome assessment, selective reporting, or other biases. One trial reported blinding of participants and personnel and incomplete outcome data.

**TABLE 1:** The baseline characteristics of included trials.

Study	Region	Sample size	Mean age (years)	Sex (male/female)	Duration of disease (years)	Treatment regimen		Dose of bailing capsule	Treatment duration
						Intervention	Control		
Zhan (2020)	Zhejiang	42/42	47.02/46.88	58/26	1.31/1.26	Bailing + Western medicine	Western medicine	0.8 g*3 times/d	3 months
Shi (2020)	Henan	46/46	58.79/59.13	51/41	0.33/0.35	Western medicine + insulin glargine + Bailing	Western medicine + insulin glargine	2 g*3 times/d	12 weeks
Zhou (2019)	Hubei	47/47	60.39/61.03	55/39	8.94/9.12	Metformin + Bailing	Metformin	3 tablets*3 times/d	12 weeks
Zong et al. (2014)	Shandong	50/50	62.48/61.22	54/46	1.39/1.43	Bailing + losartan potassium + compound α keto acid tablets	losartan potassium + compound α keto acid tablets	1 g*3 times/d	2 months
Hu et al. (2018)	Hebei	70/70	56.3/58.6	85/55	5.9/6.2	Western medicine + simvastatin + Bailing	Western medicine + simvastatin	1 g*3 times/d	3 months
Li and Gao (2021)	Hubei	58/58	55.2/55.7	61/55	5.48/5.7	Western medicine + dapagliflozin + Bailing	Western medicine + dapagliflozin	1 g*3 times/d	12 weeks
Ren and Hu (2020)	Hebei	60/60	49.8/50.3	68/52	7.3/7.7	Western medicine + simvastatin + Bailing	Western medicine + simvastatin	1 g*3 times/d	3 months
Li et al. (2016)	Guangdong	80/80	56.8/56.6	92/68	1.7/1.8	Bailing + Western medicine + liraglutide	Western medicine + liraglutide	2 tablets*3 times/d	2 months
Yang et al. (2018)	Chongqing	69/69	61.7/61.2	75/63	8.5/8.3	Bailing + Western medicine + salvia miltiorrhiza polyphenolic acid injection	Western medicine + salvia miltiorrhiza polyphenolic acid injection	3 tablets*3 times/d	4 months
Wang (2018)	Zhejiang	40/40	61.6/62.1	45/35	4.8/5.1	Bailing + alprostadil + ginkgo-damole	Alprostadil + ginkgo-damole	1 g*3 times/d	4 weeks
Tian (2019)	Henan	41/41	67.12/66.48	58/24	10.98/11.08	Western medicine + valsartan + Bailing	Western medicine + valsartan	8 tablets*3 times/d	3 months
Guan et al. (2021)	Zhejiang	42/42	51.91/52.29	54/30	9.27/9.11	Bailing + Western medicine + candesartan cilexetil	Western medicine + candesartan cilexetil	4 tablets*3 times/d	2 weeks
Luo and Hu (2011)	Henan	45/45	59.3/56.7	53/37	15.8/14.9	Western medicine + Bailing	Western medicine	1 g*3 times/d	12 weeks
Luo et al. (2018)	Fujian	31/31	53.6/54.8	27/35	3.58/4.56	Western medicine + irbesartan + Bailing	Western medicine + irbesartan	1 g*3 times/d	3 months
Hu (2016)	Shaanxi	80/80	48.7	87/73	9.3	Western medicine + irbesartan + Bailing	Western medicine + irbesartan	0.4 g*3 times/d	3 months
Jin (2016)	Zhejiang	50/50	55.3/54.1	54/46	11.9/11.5	Western medicine + valsartan + Bailing	Western medicine + valsartan	8 tablets*3 times/d	12 weeks
Gao et al. (2016)	Liaoning	31/31	53.28/55.61	34/28	-	Western medicine + Bailing	Western medicine	1–2 g*3 times/d	4 weeks
Gao (2018)	Henan	46/46	48.21/47.42	45/47	5.14/5.84	Bailing + insulin aspart	Insulin aspart	0.5 g*3 times/d	8 weeks
Zhong et al. (2020)	Hunan	57/55	59.60/58.53	65/47	3.56/3.42	Western medicine + atorvastatin calcium tablets + Bailing	Western medicine + atorvastatin calcium tablets	1 g*3 times/d	16 weeks
Wu (2018)	Henan	53/53	53.02/53.52	48/58	6.89/6.23	Western medicine + alprostadil + Bailing	Western medicine + alprostadil	4 tablets*3 times/d	2 weeks
Tang et al. (2015)	Guangdong	42/42	54.38	48/36	8.47	Western medicine + alprostadil + Bailing	Western medicine + alprostadil	4 tablets*3 times/d	2 weeks
Tang et al. (2017)	Sichuan	35/35	57.14/56.37	42/28	-	Western medicine + insulin aspart + Bailing	Western medicine + insulin aspart	1 g*3 times/d	8 weeks
Zhang (2020)	Henan	31/31	56.71/57.48	31/31	-	Valsartan + Bailing	Valsartan	1 g*3 times/d	4 months
Peng et al. (2016)	Peking	75/75	54.2/53.2	87/54	7.1/7.2	Western medicine + valsartan + Bailing	Western medicine + valsartan	6 tablets*3 times/d	3 months
Yang (2016)	Guangxi	41/41	62.8/63.1	42/40	-	Western medicine + Bailing	Western medicine	3 times/d	12 weeks
Yang (2020)	Shandong	72/70	59.04/60.34	89/53	8.50/8.80	Valsartan + Bailing	Valsartan	2 g*3 times/d	3 months
Yang and Liu (2016)	Hubei	45/45	72.3/71.4	53/37	-	Western medicine + enalapril + Bailing	Western medicine + enalapril	1 g*3 times/d	12 weeks
Lin (2016)	Liaoning	40/40	50∼70	40/40	-	Western medicine + benazepril + Bailing	Western medicine + benazepril	1 g*3 times/d	12 weeks
Niu (2020)	Henan	85/85	38.80/38.20	130/40	-	Western medicine + pancreatic kininogenase enteric-coated tablets + Bailing	Western medicine + pancreatic kininogenase enteric-coated tablets	2 g*3 times/d	8 weeks
Wang et al. (2016)	Shaanxi	50/50	68.8/69.7	61/39	2.7/2.8	Captopril + Bailing	Captopril	5 tablets*3 times/d	6 months
Wang (2011)	Liaoning	48/48	61.8/64.7	46/50	8.6/8.3	Western medicine + valsartan + Bailing	Western medicine + valsartan	2 g*3 times/d	3 months
Cheng et al. (2020)	Henan	44/44	52.32/54.01	47/41	5.72/6.32	Simvastatin + Bailing	Simvastatin	0.8 g*3 times/d	4 weeks
Dong et al. (2019b)	Qinghai	43/43	74.07/73.07	45/41	10.06/9.53	Western medicine + alprostadil + Bailing	Western medicine + alprostadil	5 tablets*3 times/d	8 weeks
Chen et al. (2016)	Guangxi	35/35	62.3/63.4	36/34	-	Western medicine + benazepril + Bailing	Western medicine + benazepril	1 g*3 times/d	12 weeks

**TABLE 2:** Quality assessment of included trials.

Study	Random sequence generation	Allocation concealment	Blinding of participants and personnel	Blinding of outcome assessment	Incomplete outcome data	Selective reporting	Other bias
Zhan (2020)	Low risk	Unclear	Unclear	Unclear	Unclear	Unclear	Unclear
Shi (2020)	Low risk	Unclear	Unclear	Unclear	Unclear	Unclear	Unclear
Zhou (2019)	Low risk	Unclear	Unclear	Unclear	Unclear	Unclear	Unclear
Zong et al. (2014)	Low risk	Unclear	Unclear	Unclear	Low risk	Unclear	Unclear
Hu et al. (2018)	Low risk	Unclear	Unclear	Unclear	Unclear	Unclear	Unclear
Li and Gao (2021)	Low risk	Unclear	Unclear	Unclear	Unclear	Unclear	Unclear
Ren and Hu (2020)	Low risk	Unclear	Unclear	Unclear	Unclear	Unclear	Unclear
Li et al. (2016)	Low risk	Unclear	Unclear	Unclear	Unclear	Unclear	Unclear
Yang et al. (2018)	Low risk	Unclear	Unclear	Unclear	Unclear	Unclear	Unclear
Wang (2018)	Low risk	Unclear	Unclear	Unclear	Unclear	Unclear	Unclear
Tian (2019)	Low risk	Unclear	Unclear	Unclear	Unclear	Unclear	Unclear
Guan et al. (2021)	Low risk	Unclear	Unclear	Unclear	Unclear	Unclear	Unclear
Luo and Hu (2011)	Low risk	Unclear	Unclear	Unclear	Unclear	Unclear	Unclear
Luo et al. (2018)	Low risk	Unclear	Unclear	Unclear	Unclear	Unclear	Unclear
Hu (2016)	Low risk	Unclear	Unclear	Unclear	Unclear	Unclear	Unclear
Jin (2016)	Low risk	Unclear	Unclear	Unclear	Unclear	Unclear	Unclear
Gao et al. (2016)	Unclear	Unclear	Unclear	Unclear	Unclear	Unclear	Unclear
Gao (2018)	Low risk	Unclear	Unclear	Unclear	Unclear	Unclear	Unclear
Zhong et al. (2020)	Low risk	Unclear	Unclear	Unclear	Unclear	Unclear	Unclear
Wu (2018)	Unclear	Unclear	Unclear	Unclear	Unclear	Unclear	Unclear
Tang et al. (2015)	Unclear	Unclear	Unclear	Unclear	Unclear	Unclear	Unclear
Tang et al. (2017)	Unclear	Unclear	Unclear	Unclear	Unclear	Unclear	Unclear
Zhang (2020)	High risk	Unclear	Unclear	Unclear	Unclear	Unclear	Unclear
Peng et al. (2016)	Unclear	Unclear	Unclear	Unclear	Unclear	Unclear	Unclear
Yang (2016)	Unclear	Unclear	Unclear	Unclear	Unclear	Unclear	Unclear
Yang (2020)	Unclear	Unclear	Unclear	Unclear	Unclear	Unclear	Unclear
Yang and Liu (2016)	Unclear	Unclear	Low risk	Unclear	Unclear	Unclear	Unclear
Lin (2016)	Unclear	Unclear	Unclear	Unclear	Unclear	Unclear	Unclear
Niu (2020)	Unclear	Unclear	Unclear	Unclear	Unclear	Unclear	Unclear
Wang et al. (2016)	Unclear	Unclear	Unclear	Unclear	Unclear	Unclear	Unclear
Wang (2011)	Unclear	Unclear	Unclear	Unclear	Unclear	Unclear	Unclear
Cheng et al. (2020)	Unclear	Unclear	Unclear	Unclear	Unclear	Unclear	Unclear
Dong et al. (2019b)	Unclear	Unclear	Unclear	Unclear	Unclear	Unclear	Unclear
Chen et al. (2016)	Unclear	Unclear	Unclear	Unclear	Unclear	Unclear	Unclear

#### Serum creatinine

**FIGURE 3:**
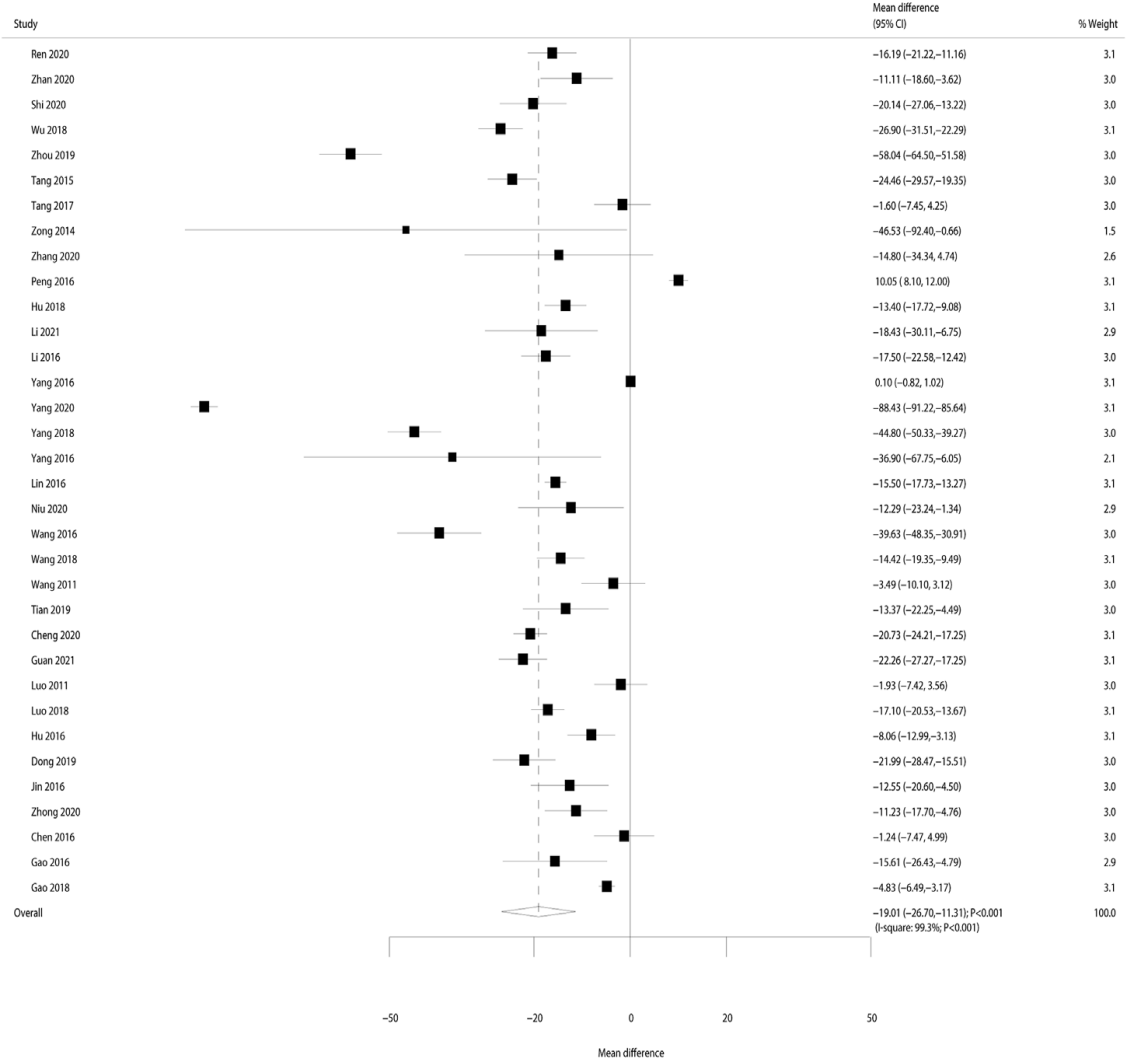
Forest plot for the effect of Bailing capsules combined with Western medicine on the change of serum creatinine as compared with Western medicine alone.

After pooling all the included studies, the heterogeneity across the included trials was substantial (*I* ^*2*^ = 99.3%; *p* < 0.001); thus, the random-effects model was applied. We noted that Bailing capsules combined with Western medicine was associated with a greater reduction in serum creatinine than Western medicine alone (weighted mean difference: −19.01 μmol/L; 95% CI: −26.70 to −11.32; *p* < 0.001; Figure 3). The results of meta-regression analyses found sample size (*p* = 0.373), mean age (*p* = 0.189), male proportion (*p* = 0.401), duration of disease (*p* = 0.937), and treatment duration (*p* = 0.575) did not contribute significant heterogeneity for the effect of Bailing capsules combined with Western medicine on serum creatinine (Supplementary Table S5).

#### Safety

Seventeen of the included trials reported adverse events; the prevalence of adverse events was lower, and the symptoms were mild (Supplementary Table S6). Three of the trials found that the risk of adverse events in the Bailing capsule group was lower than that in the control group, while five trials did not find a significant difference in the risk of adverse events between the Bailing capsule and control groups. The remaining nine trials found that adverse events did not occur during treatment.

### Economic evaluations

#### Base-case analysis

The QALYs in the Bailing capsules group and Western medicine group were 4.44 and 4.04, respectively, and Bailing capsules combined with Western medicine was associated with an increase of 0.39 QALYs as compared with Western medicine alone. The costs in the Bailing capsules and Western medicine groups were CNY 326,720 and CNY 301,999, respectively; the cost for the Bailing capsule group combined with the Western medicine group increased by CNY 24,721. The ICER for Bailing capsules was CNY 63,001 per QALY gained, which was lower than the WTP (CNY 80,976), suggesting that the use of Bailing capsules combined with Western medicine is more economical than Western medicine alone for patients with diabetic nephropathy.

#### Sensitivity analysis

**FIGURE 4:**
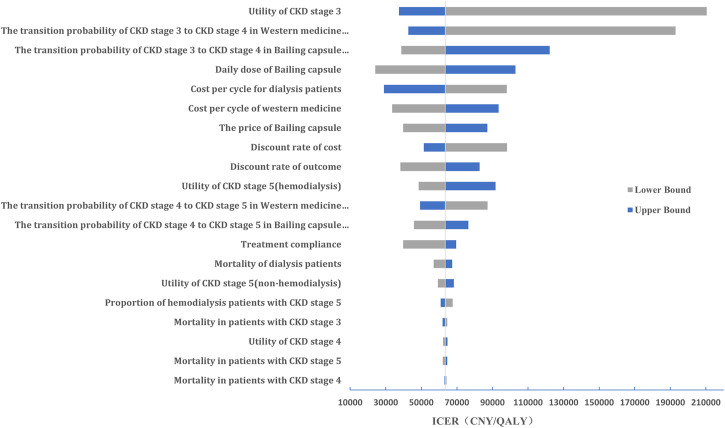
The results of the one-way sensitivity analysis.

The results of the one-way sensitivity analysis are shown in Figure 4. We noted that the results were more sensitive to changes in the CKD stage 3 utility value, transition probability from CKD stage 3 to stage 4, and daily dose of Bailing capsule.

**FIGURE 5:**
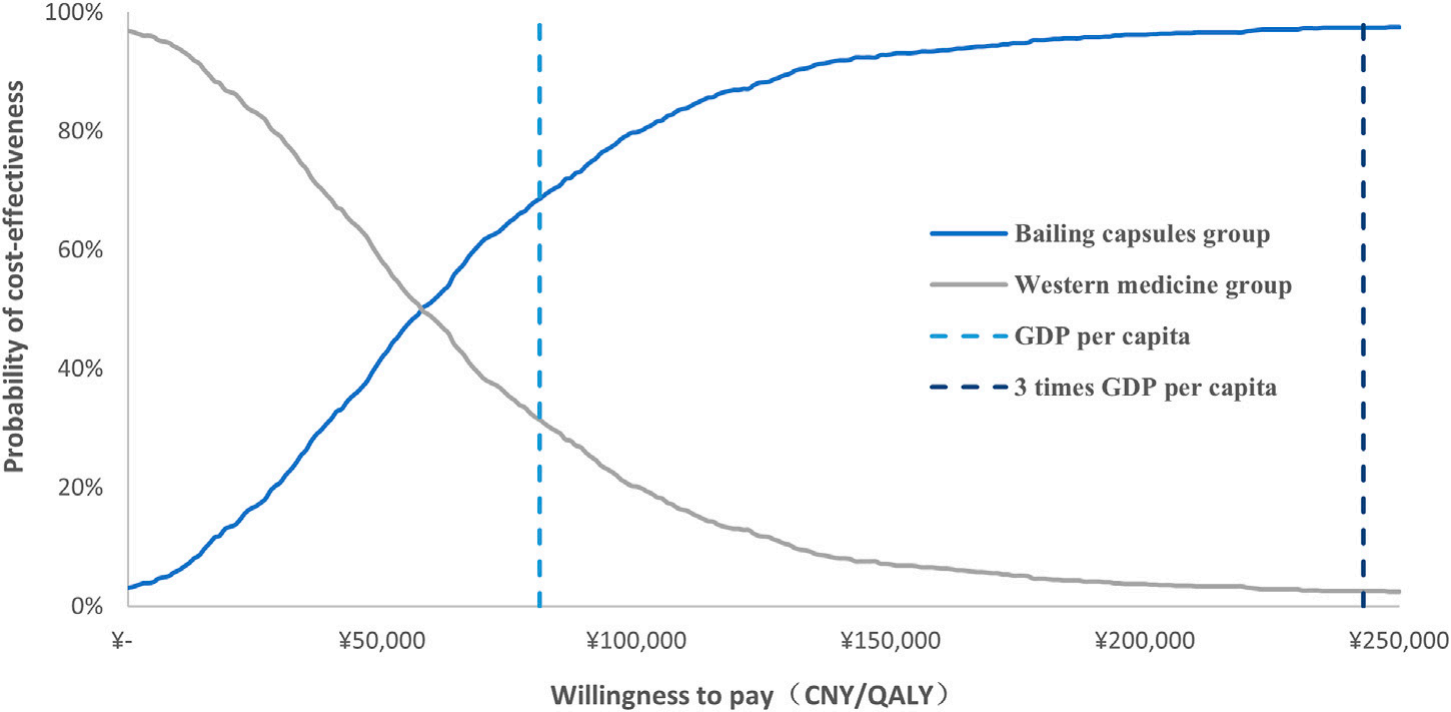
The cost-effectiveness acceptability curve after 1,000 Monte Carlo simulations.

The cost-effectiveness acceptability curve after 1,000 Monte Carlo simulations is shown in Figure 5; these data indicated that the probability of cost-effectiveness for Bailing capsules was 68.6% for WTP values corresponding to the GDP *per capita* in 2021 (CNY 80,976); the probability of cost-effectiveness for Bailing capsules increased to 97.4% for WTP values corresponding to three times the GDP *per capita* in 2021 (CNY 242,928).

## Discussion

The progression of end-stage renal disease is mainly caused by diabetic nephropathy; slowing the progression of this complication can reduce the cost of dialysis. Bailing capsules are widely used for patients with diabetic nephropathy, and studies have demonstrated their therapeutic effects (Sheng et al., 2020). However, there is a lack of consensus on the cost-effectiveness of Bailing capsules. A total of 3,444 patients in 34 RCTs were identified, with a broad range of patient characteristics. We noted that combined treatment with Bailing capsules and Western medicine was associated with a greater reduction in serum creatinine than Western medicine alone. Moreover, Bailing capsules combined with Western medicine showed an increase of 0.39 QALYs, and the cost was increased by CNY 24,721 compared to Western medicine alone. The ICER was CNY 63,001 per QALY, while 68.6% and 97.4% were likely to be cost-effective for Bailing capsules if WTP was GDP *per capita* in 2021 (CNY 80,976) and three times GDP *per capita* in 2021 (CNY 242,928), respectively.

A previous meta-analysis demonstrated the efficacy and safety of Bailing capsules for type 2 diabetic nephropathy (Sheng et al., 2020). This study identified 24 studies and found that the use of Bailing capsules was associated with an increased incidence of effective rate and greater reduction in 24 h urine protein, urine albumin excretion rate, serum creatinine, and blood urea nitrogen levels. *Cordyceps* polysaccharides and many potentially biologically active ingredients in Bailing capsules have been demonstrated to improve hormone levels, blood circulation, blood pressure, and platelet aggregation, and to exert anti-inflammatory and anti-anoxic effects (Liu and Wu, 2015). Moreover, the content in free radicals, lipid peroxides, and superoxide dismutase, and parameters such as renal blood flow, lysosomal membranes, and renal cell repair are improved by the adenosine in Bailing capsules. Thus, Bailing capsules reduce renal microvascular disease and urine albumin excretion and improve renal function (Pilemann-Lyberg et al., 2018). Glomerular sclerosis is delayed after treatment with Bailing capsules via inhibition of the proliferation of glomerular mesangial cells (Zhao et al., 2018). However, whether Bailing capsules are a cost-effective treatment choice for patients with diabetic nephropathy among the Chinese population remains controversial. Considering that the CKD stage was divided based on albuminuria, the probability of disease progression to the next stage was not available based on the reported albuminuria because the follow-up duration was shorter. In the planning stage, we intended to determine the disease status according to GFR because this parameter is used for assessing renal function in patients with CKD; however, most included trials did not report changes in GFR. Therefore, the current study used serum creatinine as an effect estimate, and the modification of diet in renal disease equation was used to calculate GFR.

In the current study, we found that Bailing capsules combined with Western medicine were associated with a greater reduction in serum creatinine compared with Western medicine alone, which was consistent with prior meta-analyses (Luo et al., 2015; Sheng et al., 2020). The probability of disease progression to the next stage could not be obtained from the included trials, because the treatment duration was shorter. Here, we estimated the transition probability between various stages of CKD via microscopic simulation of 10,000 patient cohorts and constructed a Markov model to simulate disease progression; the direct medical costs and QALYs for diabetic nephropathy were estimated over 20 years. Additionally, we found that Bailing capsules combined with Western medicine could provide an additional 0.39 QALYs than Western medicine alone, with a cost increase of CNY 24,721; thus, the ICER was CNY 63,001 per QALY gained. The ICER was lower than the WTP, which suggests that Bailing capsules combined with Western medicine are more cost-effective than Western medicine alone for treating diabetic nephropathy. One-way sensitivity analysis revealed that CKD stage 3 utility value, transition probability for CKD stage 3 to CKD stage 4, and daily dose of Bailing capsule were the top three factors affecting the results. Moreover, PSA found that at a WTP level of CNY 80,976 per QALY gained, the estimated probability of the Bailing capsule group being cost-effective was 68.6%. At CNY 242,928 per QALY, this probability was 97.4%.

This study had several limitations. First, there was substantial heterogeneity in serum creatinine levels across the included trials, which was not fully explained by the meta-regression analysis. This result might be related variable severity of diabetic nephropathy and daily dose of Bailing capsules. Second, the trial quality in most studies reported unclear risk biases for allocation concealment, blinding of participants and personnel, blinding of outcome assessment, incomplete outcome data, selective reporting, or other biases, which could affect the reliability of the pooled conclusion. Third, the current study hypothesized that the effect of Bailing capsules mainly delayed disease progression from CKD stage 3 to stage 4, while studies found that Bailing capsules could improve the micro-inflammatory status and nutritional status in hemodialysis patients; thus, medical costs could be reduced and quality of life increased (Liu et al., 2018; Zhu, 2019; Jiang et al., 2020). However, the economic value of Bailing capsules may be underestimated owing to their benefits for hemodialysis because of the absence of cost and utility data. Fourth, some transition probabilities were calculated by microsimulation based on studies with short-term follow-up owing to the long-term effect of Bailing capsules on diabetic nephropathy. Finally, the utility value and reduction in GFR were obtained based on studies not conducted in China, which could affect the cost-effectiveness of Bailing capsules for treating diabetic nephropathy in this country.

## Conclusion

Our study provided insdications that Bailing capsules combined with Western medicine are a cost-effective option compared with Western medicine alone for treating diabetic nephropathy. Further studies with a rigorous design should be performed to strengthen the findings on the cost-effectiveness of Bailing capsules.

## Data Availability

The original contributions presented in the study are included in the article/Supplementary Material, further inquiries can be directed to the corresponding author.
