# Supplementary material for: Economic evaluation of bailing capsules for patients with diabetic nephropathy in China
Source: Front Pharmacol. 2023 Jul 5;14:1175310. doi: 10.3389/fphar.2023.1175310 (PMC10354420; doi:10.3389/fphar.2023.1175310)
Supplement: Supplementary file 1 [file Table1.DOCX]

**Search strategy in PubMed:**

#1 diabetic kidney disease [Title/Abstract]

#2 DKD [Title/Abstract]

#3 diabetic nephropathy [Title/Abstract]

#4 DN [Title/Abstract]

#5 #1 OR #2 OR #3 OR #4

#6 corbrin capsule [Title/Abstract]

#7 aweto [Title/Abstract]

#8 artificial aweto preparation [Title/Abstract]

#9 cordyceps sinensis [Title/Abstract]

#10 Chinese caterpillar fungus [Title/Abstract]

#11 Chinese medicine [Title/Abstract]

#12 traditional Chinese medicine [Title/Abstract]

#13 combine traitional Chinese and western medicine [Title/Abstract]

#14 #6 OR #7 OR #8 OR #9 OR #10 OR #11 OR #12 OR #13

#15 clinical trial [Filter]

#16 randomized controlled trial [Filter]

#17 #15 OR #16

#18 #5 AND #14 AND #17
