# Supplementary material for: Economic evaluation of bailing capsules for patients with diabetic nephropathy in China
Source: Front Pharmacol. 2023 Jul 5;14:1175310. doi: 10.3389/fphar.2023.1175310 (PMC10354420; doi:10.3389/fphar.2023.1175310)
Supplement: Supplementary file 2 [file Table2.DOCX]

**CKD3**

**CKD4**

**CKD5**

**Hemodialysis**

**Death**

Figure 1. Markov model
