# Supplementary material for: Economic evaluation of bailing capsules for patients with diabetic nephropathy in China
Source: Front Pharmacol. 2023 Jul 5;14:1175310. doi: 10.3389/fphar.2023.1175310 (PMC10354420; doi:10.3389/fphar.2023.1175310)
Supplement: Supplementary file 3 [file Table5.DOCX]

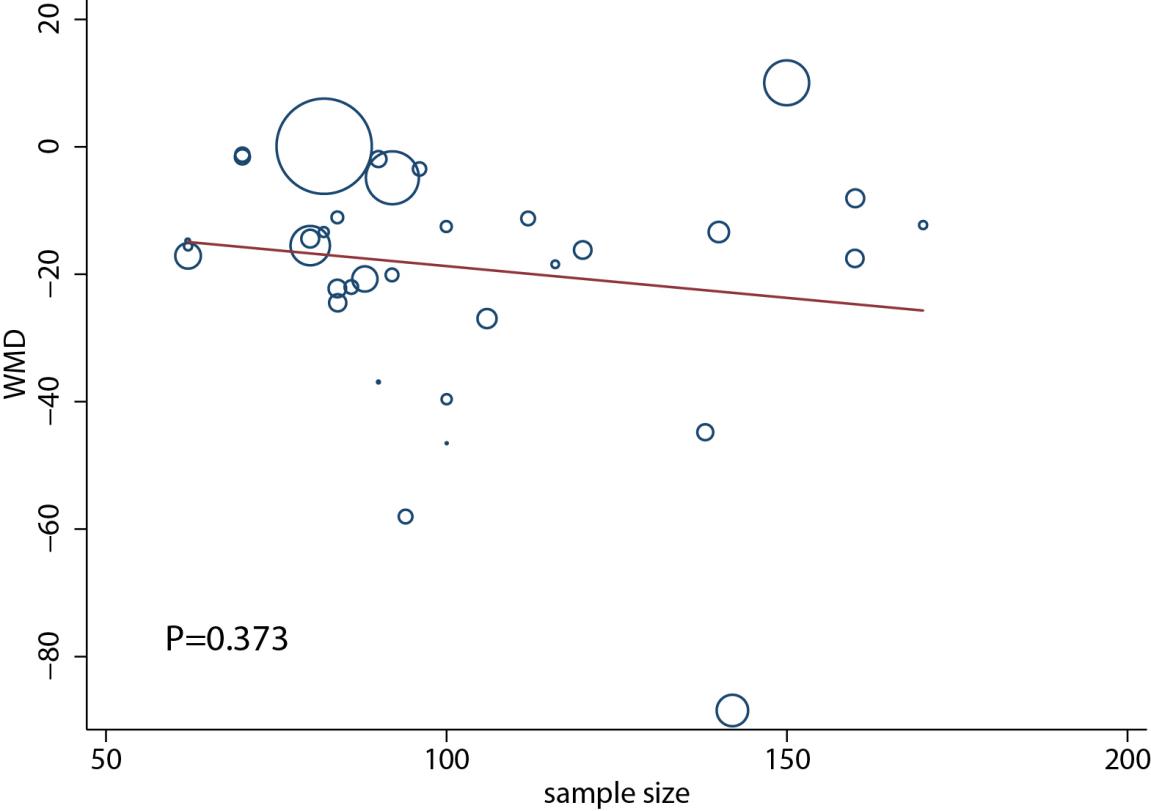


Figure 1. Meta-regression analyses based on sample size for the effect of Bailing capsule on serum creatinine


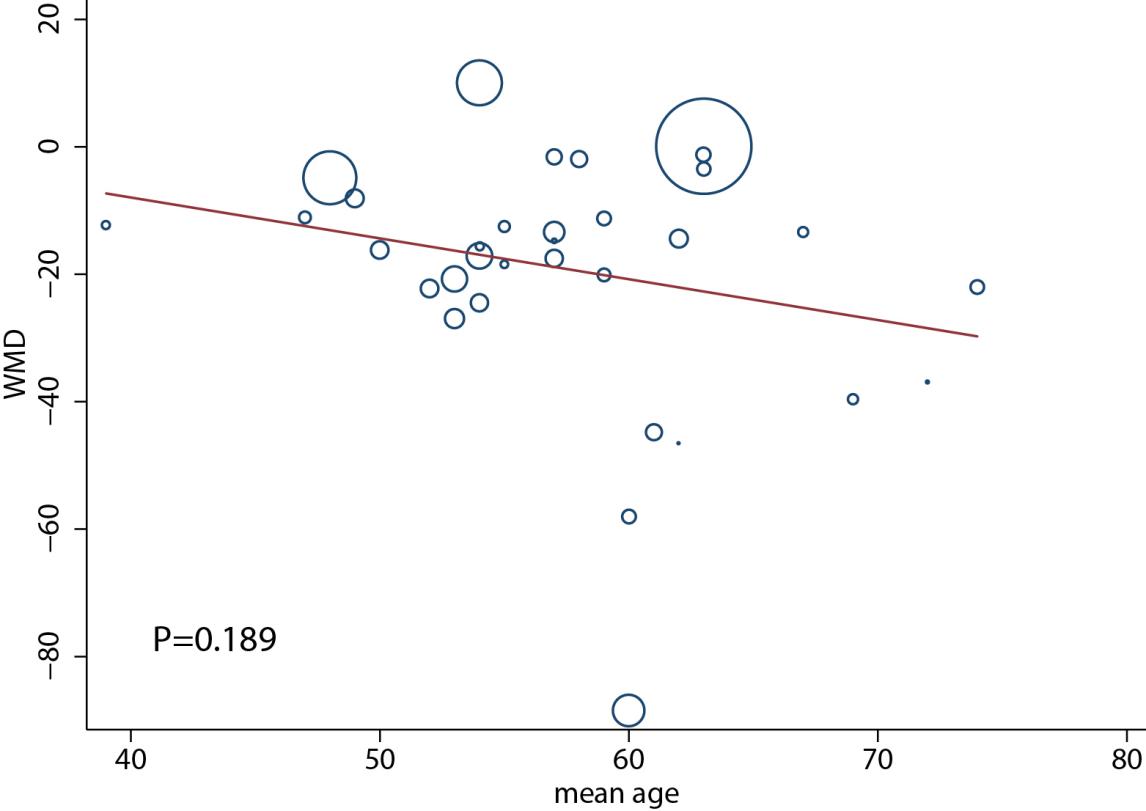


Figure 2. Meta-regression analyses based on mean age for the effect of Bailing capsule on serum creatinine


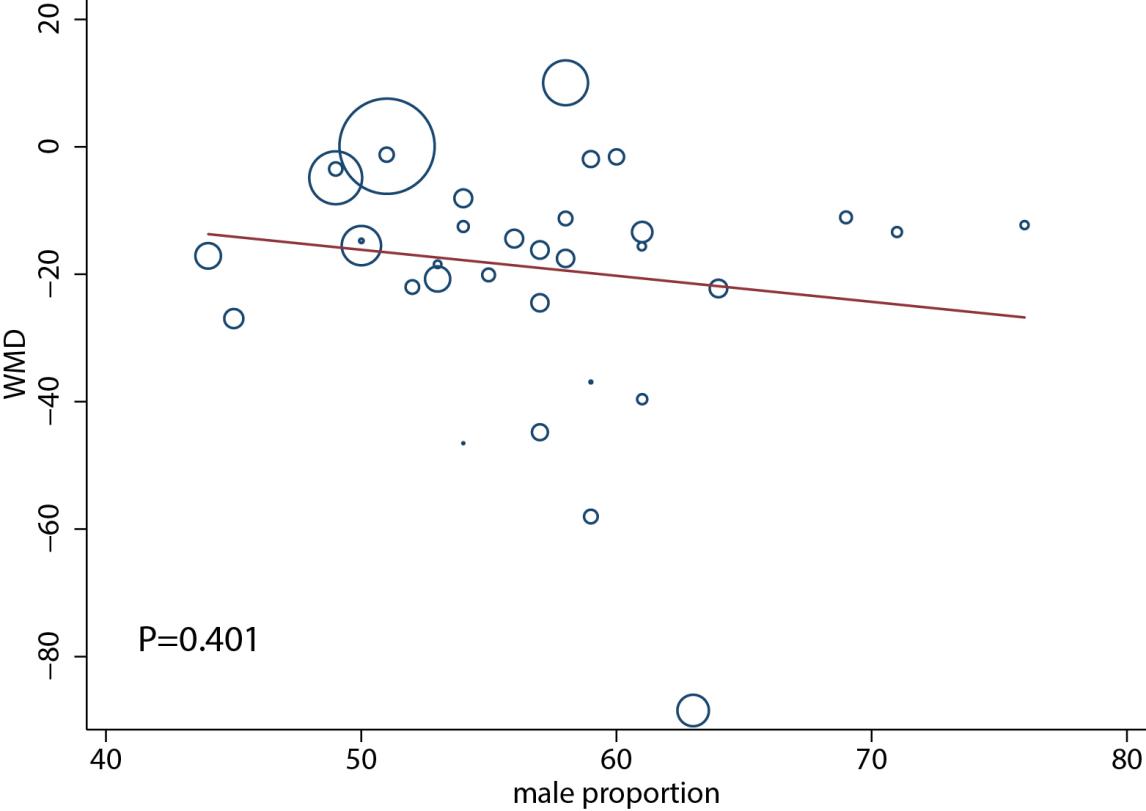


Figure 3. Meta-regression analyses based on male proportion for the effect of Bailing capsule on serum creatinine


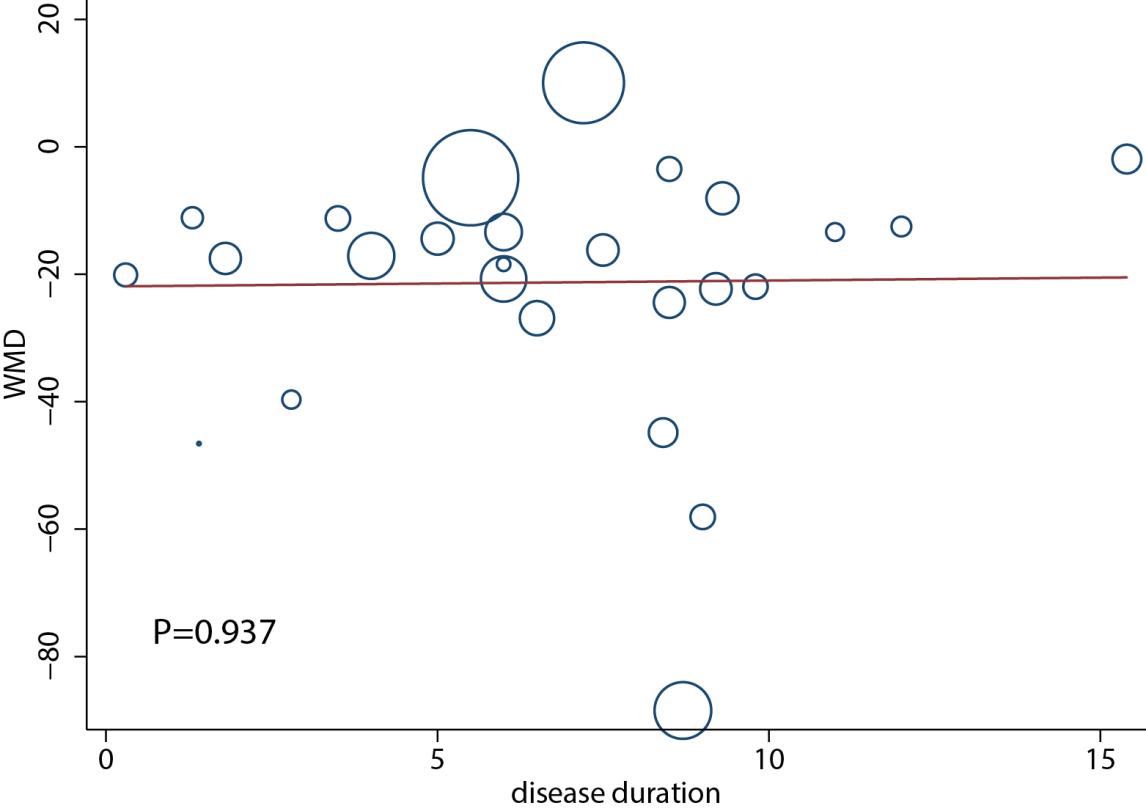


Figure 4. Meta-regression analyses based on disease duration for the effect of Bailing capsule on serum creatinine


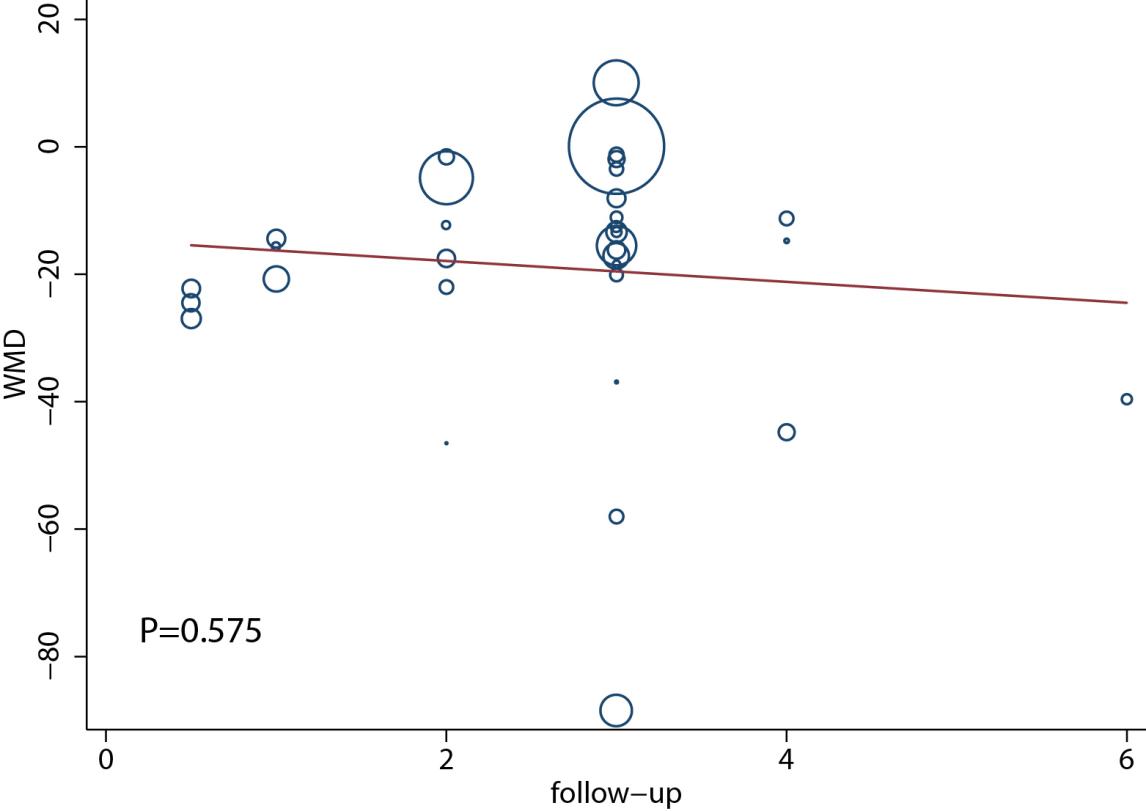


Figure 5. Meta-regression analyses based on treatment duration for the effect of Bailing capsule on serum creatinine
