# Supplementary material for: Economic evaluation of bailing capsules for patients with diabetic nephropathy in China
Source: Front Pharmacol. 2023 Jul 5;14:1175310. doi: 10.3389/fphar.2023.1175310 (PMC10354420; doi:10.3389/fphar.2023.1175310)
Supplement: Supplementary file 5 [file Table4.DOCX]

Table 1. Markov model parameters

| **Parameters** | **Base value** | **Lower limit value** | **Upper limit value** | **Distribution** | **Alpha** | **Bata** | **Source** |
| --- | --- | --- | --- | --- | --- | --- | --- |
| **Probability** |  |  |  |  |  |  |  |
| The transition probability of CKD stage 3 to CKD stage 4 in Bailing capsule group | 0.115 | 0.081 | 0.150 | Beta | 1154 | 8846 | Microsimulation simulation |
| The transition probability of CKD stage 3 to CKD stage 4 in Western medicine group | 0.197 | 0.138 | 0.256 | Beta | 1970 | 8030 | Microsimulation simulation |
| The transition probability of CKD stage 4 to CKD stage 5 in Bailing capsule group | 0.258 | 0.180 | 0.335 | Beta | 2576 | 7424 | Microsimulation simulation |
| The transition probability of CKD stage 4 to CKD stage 5 in Western medicine group | 0.324 | 0.227 | 0.422 | Beta | 3243 | 6757 | Microsimulation simulation |
| Death rate in patients with CKD stage 3 | 0.041 | 0.029 | 0.053 | Beta | 47.91 | 1120.60 | Reference [1] |
| Death rate in patients with CKD stage 4 | 0.080 | 0.056 | 0.104 | Beta | 45.92 | 528.08 | Reference [1] |
| Death rate in patients with CKD stage 5 (non-hemodialysis) | 0.118 | 0.082 | 0.153 | Beta | 44.00 | 330.17 | Reference [2] |
| Death rate in patients with CKD stage 5 (hemodialysis) | 0.085 | 0.060 | 0.111 | Beta | 45.66 | 490.88 | Reference [3] |
| Proportion of CKD stage 5 patients undergoing hemodialysis | 0.605 | 0.423 | 0.786 | Beta | 19.17 | 12.54 | Reference [4] |
| **Cost** |  |  |  |  |  |  |  |
| Unit price of Bailing capsule (CNY/0.5g*tablets) | 1.03 | 0.72 | 1.34 | Gamma | 50 | 0.02 | Reference [5] |
| Daily dose of Bailing capsule (tablets) | 12 | 9 | 18 | Uniform |  |  | 说明书 |
| Medication compliance | 0.93 | 0.65 | 1.00 | Beta | 7.26 | 0.57 | Reference [6] |
| Cost of routine treatment (CNY/year) | 34200 | 23940 | 44460 | Gamma | 50 | 684.00 | Reference [7] |
| Cost of hemodialysis (CNY/year) | 60609 | 42426 | 78792 | Gamma | 50 | 1212.18 | Reference [8] |
| **Utility value** |  |  |  |  |  |  |  |
| CKD stage 3 | 0.67 | 0.47 | 0.87 | Beta | 15.83 | 7.80 | Reference [9] |
| CKD stage 4 | 0.55 | 0.39 | 0.72 | Beta | 21.95 | 17.96 | Reference [9] |
| CKD stage 5 | 0.54 | 0.38 | 0.70 | Beta | 22.46 | 19.13 | Reference [9] |
| **Discount rate** |  |  |  |  |  |  |  |
| Cost annual discount rate | 5% | 0% | 8% | Uniform |  |  | Reference [10] |
| Outcome annual discount rate | 5% | 0% | 8% | Uniform |  |  | Reference [10] |

**Reference**

1. Sugrue DM, Ward T, Rai S, et al. Economic Modelling of Chronic Kidney Disease: A Systematic Literature Review to Inform Conceptual Model Design. Pharmacoeconomics. 2019;37(12):1451-1468.
2. Wang YF, Yang R, Bo Q. Study on factors related to death in patients with end-stage renal disease. Anhui Medical and Pharmaceutical Journal 2016; 20: 324-7.
3. Cui XB, Zou T, Tang XT, et al. Effect of Bailing capsule on survival rate and quality of life in maintenance hemodialysis patients. Asia-Pacific Traditional Medicine 2017; 13: 141-2.
4. Zhao K, Qi XR, Sui BY, et al. Analysis on budget impact of peritoneal dialysis for end-stage renal disease. Chinese Health Economics 2015;34:66-9.
5. https://www.yaozh.com/[EB/OL].
6. Chen WH, Mo YW, Wang J. Investigation on medical compliance of patients with chronic kidney disease. Journal of PLA Nursing 2011;28:8-10.
7. Guan HJ, Han S, Wang YN, et al. Pharmacoeconomic evaluation of Shenyankangfu tablets plus conventional therapy in treating diabetic nephropathy. Chin J New Drugs 2017; 26: 2491-2496.
8. Zuo L, Liu XL, Han S. Research of economic burden of CKD patients with anemia in China. Chinese Journal of Pharmacoeconomics 2018;13:11-16.
9. Gorodetskaya I, Zenios S, McCulloch CE, et al. Health-related quality of life and estimates of utility in chronic kidney disease. Kidney Int 2005;68:2801-8.
10. Liu GE, Hu SL. Guidelines for pharmacoeconomic evaluation in China (2020 edition). Chinese Journal of Pharmacoeconomics 2020
