# Supplementary material for: Economic evaluation of bailing capsules for patients with diabetic nephropathy in China
Source: Front Pharmacol. 2023 Jul 5;14:1175310. doi: 10.3389/fphar.2023.1175310 (PMC10354420; doi:10.3389/fphar.2023.1175310)
Supplement: Supplementary file 6 [file Table6.DOCX]

Table 1. The summary results of adverse events

| Zhan 2020 | Not available |
| --- | --- |
| Shi 2020 | Bailing capsule group: Hypoglycemia 6.52%, pharyngeal discomfort 4.35%, malnutrition 2.17%, the total incidence of 13.04%; Control group: hypoglycemia 4.35%, pharyngeal discomfort 0.00%, malnutrition 2.17%, the total incidence of 6.52% |
| Zhou 2019 | Not available |
| Zong 2014 | No serious adverse events |
| Hu 2018 | Bailing capsule group: 2 rash, 1 pharyngeal discomfort, 5 gastrointestinal discomfort, 1 pharyngeal discomfort, and 3 abnormal liver function, with a total incidence of 17.1%; Control group: 1 rash, 1 pruritus, 5 gastrointestinal discomfort, and 2 abnormal liver function, with a total incidence of 12.9% |
| Li 2021 | Bailing capsule group: 9 adverse reactions (15.52%), including 7 hypoglycemia and 2 hypotension; Control group: 11 adverse reactions (18.97%), including 9 hypoglycemia and 2 hypotension |
| Ren 2020 | No serious adverse events |
| Li 2016 | Bailing capsule group: 3 patients with hypoglycemia; Control group: 12 patients with hypoglycemia |
| Yang 2018 | Not available |
| Wang 2018 | Not available |
| Tian 2019 | No serious adverse events |
| Guan 2021 | Bailing capsule group: Nausea and vomiting 4.76%, dizziness 2.38%, the total incidence of adverse reactions 7.14%; Control group: Nausea and vomiting 11.90%, dyspepsia 2.22%, headache 4.76%, the total incidence of adverse reactions 19.05% |
| Luo 2011 | No serious adverse events |
| Luo 2018 | Not available |
| Hu 2016 | Not available |
| Jin 2016 | Not available |
| Gao 2016 | Not available |
| Gao 2018 | Not available |
| Zhong 2020 | Bailing capsule group: 3 headache, 1 nausea and vomiting, and 1 pharyngeal discomfort, the total incidence of adverse reactions was 8.8%; Control group: 2 headache, 2 nausea and vomiting, 1 fever, the total incidence of adverse reactions was 9.1%. |
| Wu 2018 | Not available |
| Tang 2015 | Not available |
| Tang 2017 | Not available |
| Zhang 2020 | No serious adverse events |
| Peng 2016 | Control group: 4 cough |
| Yang 2016 | Not available |
| Yang 2020 | Bailing capsule group: 1 headache and dizziness and 1 nausea, with a total incidence of 2.78%; Control group: 6 headache and dizziness, 5 nausea and 2 pharyngeal discomfort, with a total incidence of 18.75% |
| Yang 2016 | Not available |
| Lin 2016 | Bailing capsule group: 3 nausea and vomiting |
| Niu 2020 | Bailing capsule group: 2 diarrhea; Control group: 2 nausea |
| Wang 2016 | No serious adverse events |
| Wang 2011 | Not available |
| Cheng 2020 | Not available |
| Dong 2019 | Not available |
| Chen 2016 | Bailing capsule group: 5.71% of nausea and vomiting; Control group: 8.57% of nausea and vomiting |
